# Peer Review of “Seroprevalence of SARS-CoV-2 in Niger State: Pilot Cross-Sectional Study”

**DOI:** 10.2196/50391

**Published:** 2023-10-17

**Authors:** Nadège Bourgeois-Nicolaos

**Affiliations:** 1 Department of Bacteriology-Hygiene, APHP Université Paris-Saclay, Antoine Beclere Hospital, Clamart, France; 2 Institute for Integrative Biology of the Cell, Gif-Sur-Yvette, France

**Keywords:** COVID-19, pandemic, SARS-CoV-2, seroprevalence, serology, epidemiology, Niger State, Nigeria, COVID-19 testing, social distancing

*This is a peer-review report submitted for the paper “Seroprevalence of SARS-CoV-2 in Niger State: Pilot Cross-Sectional Study.”*

## Review Round 1

### General Comments

This article is a pilot study [1] that was conducted to determine the prevalence, patterns, and dynamics of COVID-19 and the risk factors for contracting the disease in Niger State from June 26 to 30, 2020.

This study is a cross-sectional study and uses a clustered, stratified random sampling method. Only 185 participants were included in the study. The sample size is small.

The seroprevalence of COVID-19 was found to be 25.4% and 2.16% for the positive IgG and IgM, respectively. These seroprevalence results mean that herd immunity to COVID-19 has yet to be achieved, and the population is still susceptible to more infection and transmission of the virus.

### Specific Comments

#### Major Comments

1. Samples were taken randomly from 185 participants for COVID-19 IgG and IgM rapid tests and questionnaires. Information on the number of patients included in the different sampling points is missing. Have serology results been confirmed by other techniques?
2. The results are expressed as a percentage; it would be interesting to have the data on the number of samples or the number of patients.How many participants tested positive for only IgG and for both IgG and IgM?

1. Bibliographic references are not formatted in the correct format.

#### Minor Comments

1. Page 1: explain “NCDC”
2. Page 5: italicize *Chlamydia pneumoniae*, *Mycoplasma pneumoniae*, *Treponema pallidum*
3. Page 9: add percent majority (61.62%)
4. Page 11: explain “ATM”
5. Page 14: replace igM with IgG, “while the Kit detecting only IgM means that...”
6. Page 19: explain “PPE”

## Review Round 2

### General Comments

This paper describes the seroprevalence of COVID-19 in Niger State. This is a pilot study.

Despite the authors’ efforts to respond specifically to comments, some points are still missing.

### Specific Comments

#### Major Comments

1. Please include more quantitative results in the abstract (odds ratio with CIs).
2. The relative results (percentage) are not well presented. Mostly, if n is less than 100, do not use decimal points in your percentages. We need to review the data in Table 2.
3. The 95% CIs for the odds ratios are missing in Table 2.
4. The SARS-CoV-2 script must be homogenized throughout the manuscript.
5. The meaning of the a is missing in Table 2.
6. Almost all the information in Table 2 is already in the text.

#### Minor Comments

1. Page 3: replace “COVI-19” with “COVID-19” and remove “Coronavirus disease 2019”
2. The SARS-CoV-2 script must be homogenized throughout the manuscript
3. How were the kits validated by polymerase chain reaction?
4. Page 17: explain “ATMs” in the paper
